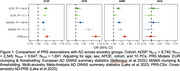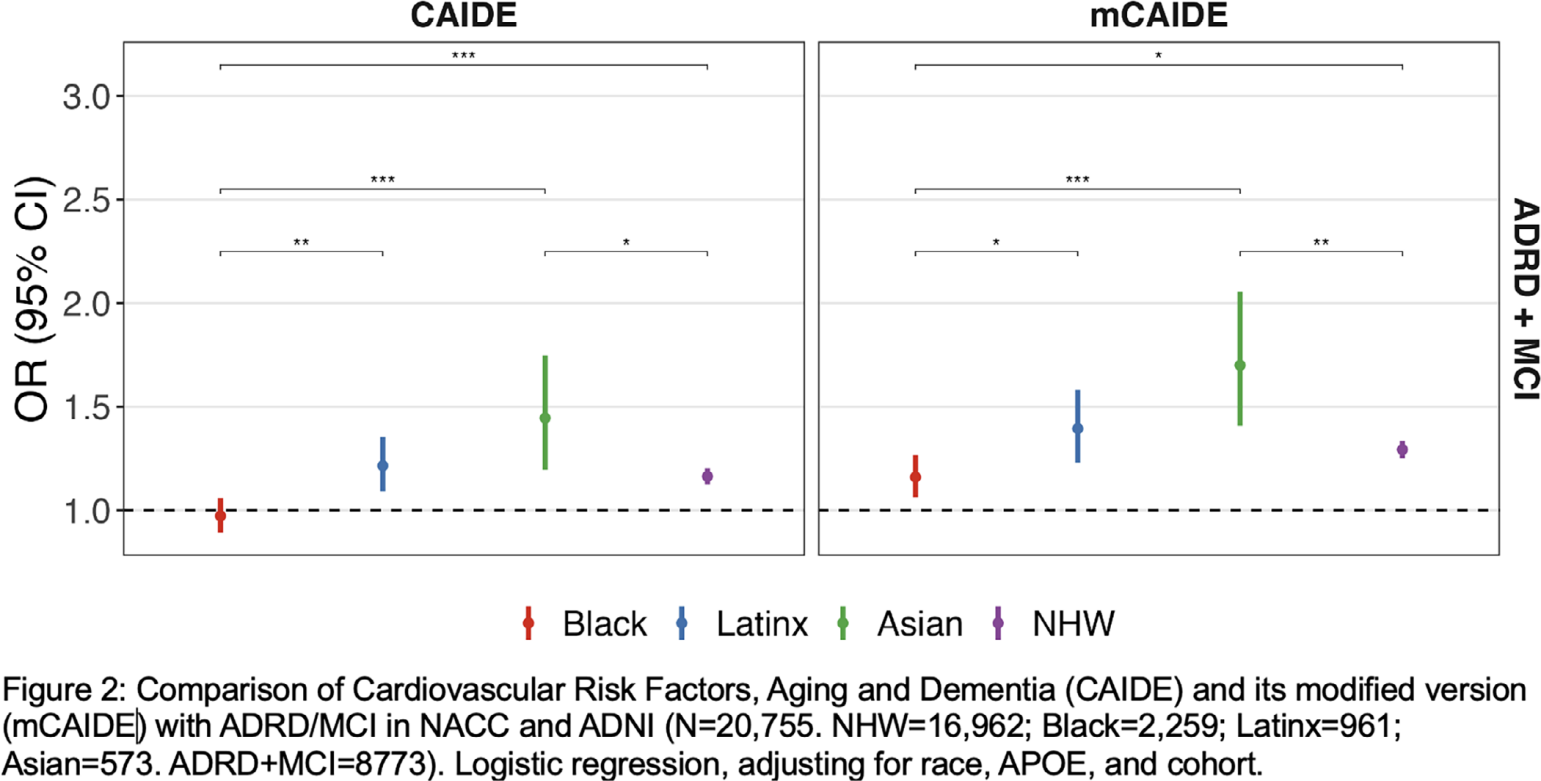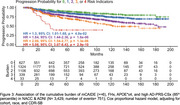# Genomic‐informed dementia risk reports for equitable personalized medicine to promote brain health

**DOI:** 10.1002/alz70855_107015

**Published:** 2025-12-24

**Authors:** Shea J Andrews, Meri Okorie, Ana I Boeriu, Caroline Jonson, Brian Fulton‐Howard, Alan E. Renton, Jennifer S. Yokoyama, Kristine Yaffe

**Affiliations:** ^1^ University of California, San Francisco, San Francisco, CA, USA; ^2^ Icahn School of Medicine at Mount Sinai, New York, NY, USA

## Abstract

**Background:**

Genomic‐informed dementia risk reports (GIDRR) that assess genetic and environmental factors can enhance Alzheimer's disease (AD) risk prediction and prevention. However, most risk models are derived from non‐Latinx White (NLW) populations and lack validation in diverse populations. Here, we evaluate the predictive accuracy of AD clinical and polygenic risk scores (CRS & PRS) in diverse populations and examine their combined effect with family history (FHx) and *APOE* on incident dementia.

**Methods:**

In ADSP (*N* = 18,623; 42% 1KG‐EUR‐like, 25% 1KG‐AMR‐like, 21% 1KG‐AFR‐like, 8.6% 1KG‐SAS‐like, and 0.2% 1KG‐EAS‐like genetic ancestry groups) we constructed three AD‐PRS (excluding *APOE*): 1KG‐EUR‐like single‐ancestry using clumping and thresholding (CT); multi‐ancestry using CT; and cross‐ancestry using PRS‐CSx‐auto. Logistic regression assessed AD‐PRS associations with AD, adjusting for age, sex, *APOE*, and PCs. In NACC & ADNI (*N* = 20,755; 82% NLW, 11% Black, 4.6% Latinx, and 2.8% Asian reported populations), we constructed two CRS—the CAIDE score and its modified version (mCAIDE)—comprising age, gender, education, hypertension, obesity, and dyslipidemia. Logistic regression assessed CRS associations with MCI/AD. Finally, we constructed a GIDRR composed of mCAIDE, FHx, APOE*ε4, and high AD‐PRS‐CSx. Cox‐proportional hazard models estimated risk of dementia progression due to increasing risk burden, adjusting for cohort, race/ethnicity, and CDR.

**Results:**

In 1KG‐EUR‐like participants, all PRS models predicted higher AD risk, whereas PRS were not predictive in 1KG‐SAS‐like participants (Figure 1). In 1KG‐AFR‐like and 1KG‐AMR‐like participants the 1KG‐EUR‐like AD‐PRS was not associated with AD; however, multi‐ancestry AD‐PRS and PRS‐CSx were predictive of AD risk. Higher CAIDE scores were associated with increased odds of MCI/AD in Asian, Latinx, and NLW participants, but not in Black participants (Figure 2). mCAIDE scores were significantly associated with increased MCI/AD risk in all groups, with the strongest associations in Asian participants and decreasing magnitude through Latinx, NLW, and Black participants. GIDRR analysis found having one high‐risk indicator increased AD risk by 27%, two indicators by 83%, three indicators doubled the risk, and four indicators led to a fivefold increase (Figure 3).

**Conclusion:**

AD PRS and CRS derived using models with consideration of genetic ancestry and race/ethnicity improve transportability and predictive accuracy. Genomic‐informed risk reports can support equitable, personalized dementia prevention strategies.